# Single-Cell Analyses of Human Eosinophils at High Resolution to Understand Compartmentalization and Vesicular Trafficking of Interferon-Gamma

**DOI:** 10.3389/fimmu.2018.01542

**Published:** 2018-07-09

**Authors:** Lívia A. S. Carmo, Kennedy Bonjour, Lisa A. Spencer, Peter F. Weller, Rossana C. N. Melo

**Affiliations:** ^1^Laboratory of Cellular Biology, Department of Biology, Federal University of Juiz de Fora, Juiz de Fora, Brazil; ^2^Department of Medicine, Beth Israel Deaconess Medical Center, Harvard Medical School, Boston, MA, United States

**Keywords:** cytokines, cell activation, degranulation, inflammation, immunonanogold electron microscopy, eosinophil, leukocytes, interferon-gamma

## Abstract

Human eosinophils release numerous cytokines that are pre-synthesized and stored within their cytoplasmic-specific (secretory) granules. For example, high levels of interferon-gamma (IFN-γ) are constitutively expressed in these cells, but the intracellular compartments involved in the transport and release of this cytokine remain to be established. In this work, we used a single-cell approach to investigate the subcellular localization of IFN-γ in human eosinophils stimulated or not with tumor necrosis factor alpha (TNF-α) or CC-chemokine ligand 11 CCL11 (eotaxin-1), inflammatory mediators that induce eosinophil activation and secretion. A pre-embedding immunonanogold transmission electron microscopy (TEM) technique that combines optimal epitope preservation and access to membrane microdomains was applied to detect precise localization of IFN-γ in combination with computational quantitative analyses. In parallel, degranulation processes and formation of eosinophil sombrero vesicles (EoSVs), large transport carriers involved in the transport of granule-derived cytokines, were investigated. Quantitative TEM revealed that both CCL11 and TNF-α-activated eosinophils significantly increased the total number of EoSVs compared to the unstimulated group, indicating that this vesicular system is actively formed in response to cell activation. Ultrastructural immunolabeling identified a robust pool of IFN-γ on secretory granules in both unstimulated and stimulated cells. Moreover, EoSVs carrying IFN-γ were seen around or/and in contact with secretory granules and also distributed in the cytoplasm. Labeling was clearly associated with EoSV membranes. The total number of IFN-γ-positive EoSVs was significantly higher in stimulated compared to unstimulated cells, and these labeled vesicles had a differential distribution in the cytoplasm of activated cells, being significantly higher in the cell periphery compared with the inner cell, thus revealing intracellular IFN-γ mobilization for release. IFN-γ extracellular labeling was found at the cell surface, including on extracellular vesicles. Our results provide direct evidence that human eosinophils compartmentalize IFN-γ within secretory granules and identify, for the first time, a vesicular trafficking of IFN-γ associated with large transport carriers. This is important to understand how IFN-γ is trafficked and secreted during inflammatory responses.

## Introduction

Eosinophils are terminally differentiated cells of the innate immune system with a broad distribution in tissues and varied functions related to both immune homeostasis and immunity [reviewed in Ref. (1–3)]. Eosinophils are sources of cytokines that are mostly stored as preformed pools within secretory (specific) granules, a robust population of large and morphologically distinctive granules, existent in the eosinophil cytoplasm [reviewed in Ref. (4)]. By mobilizing intracellular stores of preformed cytokines, human eosinophils have the capability of immediate release of these immune mediators in response to cell activation without the necessity for *de novo* synthesis [reviewed in Ref. (4–6)].

Human eosinophils are equipped with an arsenal of pre-formed Th1, Th2, and regulatory cytokines [reviewed in Ref. (4–6)]. The capacity and significance of these innate immune granulocytes to secrete specific cytokines have been recognized for mediating diverse immune-related responses. Interferon-gamma (IFN-γ), a cytokine that acts as both an inducer and a regulator for inflammation (7, 8), is one major product of human eosinophils (9). In a previous work, we showed that high levels of this Th1-associated cytokine are constitutively expressed in human circulating eosinophils and that IFN-γ signals, detected after subcellular fractionation, colocalize in granule-enriched fractions as well as in lighter cytoplasmic fractions (9). However, the intracellular compartments involved in the transport and release of this cytokine remain to be established.

Because vesicular transport of products from secretory granules underlies secretion in eosinophils and other leukocytes, a challenge to comprehend this secretory pathway has been the identification of granule-originated products in vesicular compartments [reviewed in Ref. (10)]. Our group has been using a pre-embedding immunonanogold electron microscopic technique to understand the cellular mechanisms involved in the trafficking and release of immune mediators from human eosinophils activated by inflammatory stimuli (11–16). Application of this single-cell technique, which combines several strategies for ultrastructure and antigen preservation and improved antibody penetration for detecting molecules at subcellular compartments and membrane microdomains (17), has been providing substantial insights into eosinophil content of immune mediators and their compartmentalization [reviewed in Ref. (10)].

In the present work, we used this approach to understand the intracellular distribution and trafficking of IFN-γ at a single-cell level within human eosinophils stimulated or not with inflammatory stimuli, which are recognized to induce eosinophil activation and secretion: the CC-chemokine ligand 11 CCL11 (eotaxin-1) and tumor necrosis factor alpha (TNF-α) (9, 18–21).

We found that IFN-γ is compartmentalized not only in secretory granules but also in eosinophil sombrero vesicles (EoSVs), large, granule-derived tubular carriers, typical of human eosinophils (22). An active transport of IFN-γ associated with EoSVs was identified in response to eosinophil activation.

## Materials and Methods

### Eosinophil Isolation, Stimulation, and Viability

Granulocytes were isolated from peripheral blood of allergic or healthy donors. Eosinophils were enriched and purified by negative selection using the human eosinophil enrichment cocktail (SSep™, StemCell Technologies, Seattle, WA, USA) and the MACS bead procedure (Miltenyi Biotec, Auburn, CA, USA), as previously described (23) with the exception that hypotonic red blood cell (RBC) lysis was omitted to avoid any potential for RBC lysis to affect eosinophil function. Eosinophil viability and purity were greater than 99% as determined by ethidium bromide (Molecular Probes, Life Technologies, Carlsbad, CA, USA) incorporation and cytocentrifuged smears stained with HEMA 3 stain kit (Fisher Scientific, Medford, MA, USA), respectively. Purified eosinophils (10^6^ cells/mL) were stimulated with TNF-α (200 ng/mL; R&D Systems, Minneapolis, MN, USA) or recombinant human CCL11 (100 ng/mL; R&D Systems), in RPMI-1640 medium plus 0.1% ovalbumin (Sigma, St. Louis, MO, USA), or medium alone at 37°C, for 1 h. At these concentrations, CCL11 and TNF-α induce consistent cell secretion (16).

### Ethics Statement

This study was carried out in accordance with the ethical principles taken from the Declaration of Helsinki and written informed consent was obtained from donors. Institutional Review Board (IRB) approval was obtained from the Beth Israel Deaconess Medical Center Committee on Clinical Investigation (Boston, MA, USA).

### Antibody Reagents

Mouse anti-human IFN-γ (clone B27, catalog number 554699) and irrelevant isotype control monoclonal antibodies (BD-Pharmingen, San Diego, CA, USA) were used for the ultrastructural immunodetection studies at concentrations of 5 µg/mL. The secondary Ab for immunoEM was an affinity-purified goat anti-mouse Fab fragment conjugated to 1.4-nm gold particles (1:100, Nanogold, Nanoprobes, Stony Brook, NY, USA).

### Conventional Transmission Electron Microscopy (TEM)

For conventional TEM, isolated eosinophils were prepared as before (11, 24). Cells were fixed in a mixture of freshly prepared aldehydes (1% paraformaldehyde and 1.25% glutaraldehyde) in 0.1 M sodium cacodylate buffer for 1 h at room temperature (RT), embedded in 2% agar, and kept at 4°C for further processing. Agar pellets containing eosinophils were post-fixed in 1% osmium tetroxide in a sym-collidine buffer, pH 7.4, for 2 h at RT. After washing with sodium maleate buffer, pH 5.2, pellets were stained en bloc in 2% uranyl acetate in 0.05 M sodium maleate buffer, pH 6.0 for 2 h at RT, and washed in the same buffer as before prior to dehydration in graded ethanols and infiltration and embedding with a propylene oxide-Epon sequence (Eponate 12 Resin; Ted Pella, Redding, CA, USA). After polymerization at 60°C for 16 h, thin sections were cut using a diamond knife on an ultramicrotome (Leica, Bannockburn, IL, USA). Sections were mounted on uncoated 200-mesh copper grids (Ted Pella) before staining with lead citrate and viewed with a transmission electron microscope (Tecnai Spirit G2, FEI/Thermo Fisher Scientific, Eindhoven, The Netherlands) at 60 kV.

### Cell Preparation for Immunonanogold Electron Microscopy (immunoEM)

For immunoEM, purified eosinophils were immediately fixed in fresh 4% paraformaldehyde in phosphate-buffered saline (PBS) (0.02 M sodium phosphate buffer, 0.15 M sodium chloride, pH 7.4) (17). Cells were fixed for 30 min at RT, washed in PBS and centrifuged at 1,500 *g* for 1 min. Samples were then resuspended in molten 2% agar in PBS and quickly recentrifuged. Pellets were immersed in 30% sucrose in PBS overnight at 4°C, embedded in OCT compound (Miles, Elkhart, IN, USA), and stored in −180°C liquid nitrogen for subsequent use.

### Pre-Embedding Immunonanogold EM

As detailed before (17), pre-embedding immunolabeling was carried out before standard EM processing (postfixation, dehydration, infiltration, resin embedding, and resin sectioning). All labeling steps were carried out at RT on cryosections as before (17) as follows: (a) one wash in 0.02 M PBS, pH 7.4, 5 min; (b) immersion in 50 mM glycine in 0.02 M PBS, pH 7.4, 10 min; (c) incubation in a mixture of PBS and BSA (PBS-BSA buffer; 0.02 M PBS plus 1% BSA) containing 0.1% gelatin (20 min) followed by PBS-BSA plus 10% normal goat serum (NGS) (30 min)—(this step is crucial to block non-specific Ab-binding sites); (d) incubation with primary Ab (1 h); (e) blocking with PBS-BSA plus NGS (30 min); (f) incubation with secondary Ab (1 h); (g) washing in PBS-BSA (three times of 5 min each); (h) postfixation in 1% glutaraldehyde (10 min); (i) five washings in distilled water; (j) incubation with HQ silver enhancement solution in a dark room according to the manufacturer’s instructions (Nanoprobes) (10 min). This step enables a nucleation of silver ions around gold particles. These ions precipitate as silver metal and the particles grow in size facilitating observation under TEM; (k) three washings in distilled water; (l) immersion in freshly prepared 5% sodium thiosulfate (5 min); (m) postfixation with 1% osmium tetroxide in distilled water (10 min); (n) staining with 2% uranyl acetate in distilled water (5 min); (o) embedding in Eponate (Eponate 12 Resin; Ted Pella); (p) after polymerization at 60°C for 16 h, embedding was performed by inverting eponate-filled plastic capsules over the slide-attached tissue sections; and (q) separation of eponate blocks from glass slides by brief immersion in liquid nitrogen. Thin sections were cut using a diamond knife on an ultramicrotome (Leica). Sections were mounted on uncoated 200-mesh copper grids (Ted Pella) before staining with lead citrate and viewed with a transmission electron microscope (CM 10; Philips) at 60 kV. Two controls were performed: (1) primary Ab was replaced by an irrelevant Ab and (2) primary Ab was omitted. Electron micrographs were randomly taken at different magnifications to study the entire cell profile and subcellular features.

### Quantitative EM Analysis

For immunonanogold EM quantitative studies, electron micrographs randomly taken from unstimulated and stimulated eosinophils were evaluated. A total of 93 electron micrographs (29 from unstimulated, 34 from CCL11-stimulated, and 30 from TNF-α-stimulated cells) and 4,095 secretory granules (1,260 from unstimulated, 1,499 from CCL11-stimulated, and 1,336 from TNF-α-stimulated eosinophils) were evaluated and the numbers of labeled and non-labeled granules were counted.

Additionally, the total number of EoSVs and the numbers of EoSVs positive for IFN-γ were quantitated in two cytoplasmic areas: peripheral cytoplasm (within 1.0 µm of the plasma membrane), and within the inner cytoplasm (the contiguous cytoplasmic area deeper in the cell). These analyses were done in clear cross-cell sections (total of 30 cells, *n* = 1,357 EoSVs) exhibiting the entire eosinophil cell profile, intact plasma membranes and nuclei as previously performed for single-cell analyses at a high resolution of immunogold-labeled cells (16). All quantitative studies were done using the *Image J* software *(*National Institutes of Health, Bethesda, MD, USA).

### Statistical Analyses

ANOVA followed by Turkey multiple comparisons test, or Kruskal–Wallis test was performed using GraphPad Prism version 7.00 for Windows (GraphPad Software, La Jolla, CA, USA, www.graphpad.com). Significance was *P* < 0.05.

## Results

### Eosinophil Activation Leads to Degranulation and Formation of Large Tubular Carriers

**Figure 1:**
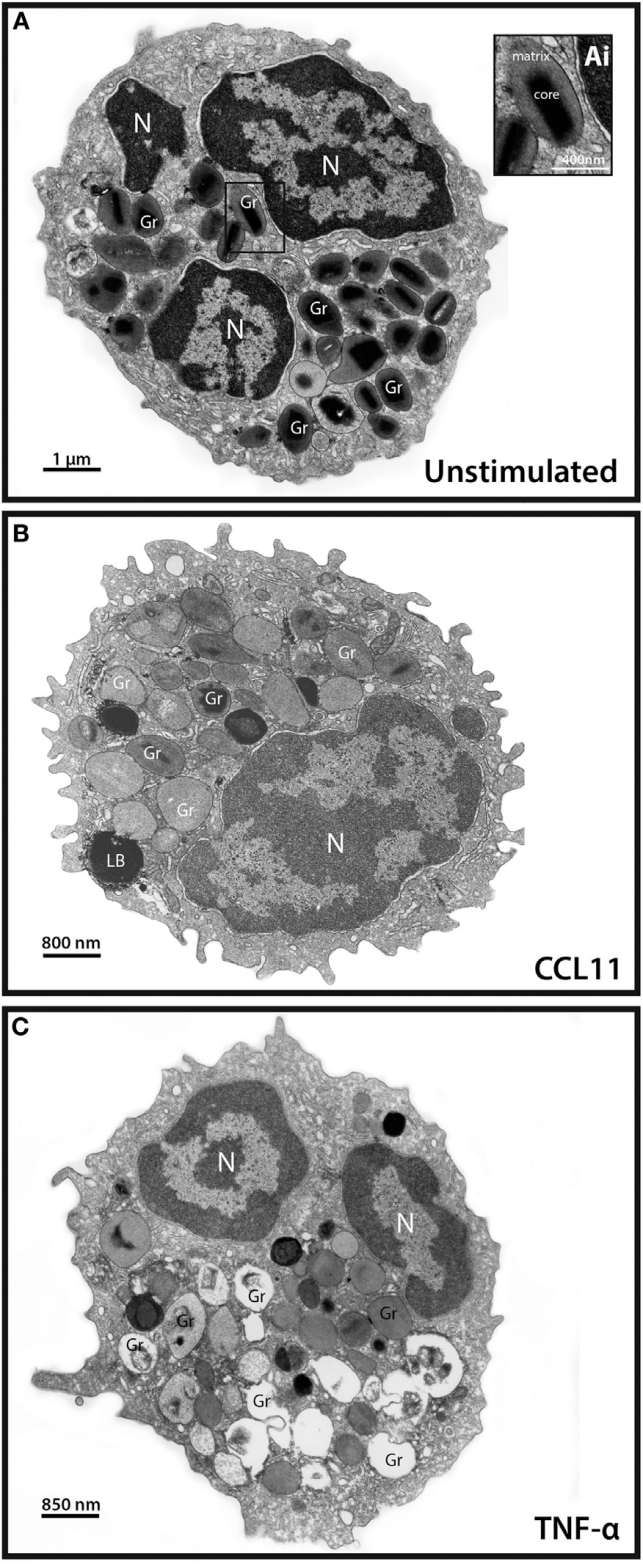
Conventional transmission electron microscopy (TEM) of resting and activated human eosinophils. **(A)** A representative eosinophil shows its typical cytoplasmic population of specific granules (Gr) with a unique morphology—an internal well-defined electron-dense crystalline core and an outer electron-lucent matrix [seen at higher magnification in (Ai)]. **(B,C)** In response to stimulation of eosinophils, their secretory granules undergo structural changes indicative of degranulation. CCL11 **(B)** induces granule content release in the absence of granule fusions while tumor necrosis factor alpha (TNF-α) **(C)** leads to fusions between secretory granules. Disarrangement of the crystalline cores is observed in stimulated **(B,C)** compared to unstimulated **(A)** cells. Note in **(A,C)**, the typical bilobed nucleus (N). Eosinophils were isolated by negative selection from healthy donors, stimulated or not with CCL11 or TNF-α for 1 h, immediately fixed and prepared for TEM.

Degranulation events in different types of secretory cells, including cells from the immune system, can be observed by means of single-cell analyses through TEM that clearly shows secretory granules exhibiting losses of contents in activated cells [reviewed in Ref. (4)]. As expected and documented before for CCL11 (11, 16) and TNF-α (16), these stimuli led to granule mobilization and content release (Figure 1). While resting eosinophils showed most granules with typical ultrastructure, that is, with an electron-dense, crystalline core in their equatorial region embedded in a less dense matrix, delimited by a typical membrane (Figure 1A), activated, degranulating eosinophils exhibited granules with ultrastructural features indicative of cell secretion [reviewed in Ref. (4, 25)]. CCL11 led to emptying of granules with morphological features of piecemeal degranulation such as enlargement and reduced electron-density of secretory granules in the absence of granule fusions (Figure 1B) (11, 25) while TNF-α triggered compound exocytosis, characterized by fusion of a number of granules with each other forming large channels in the cytoplasm (Figure 1C) (16).

**Figure 2:**
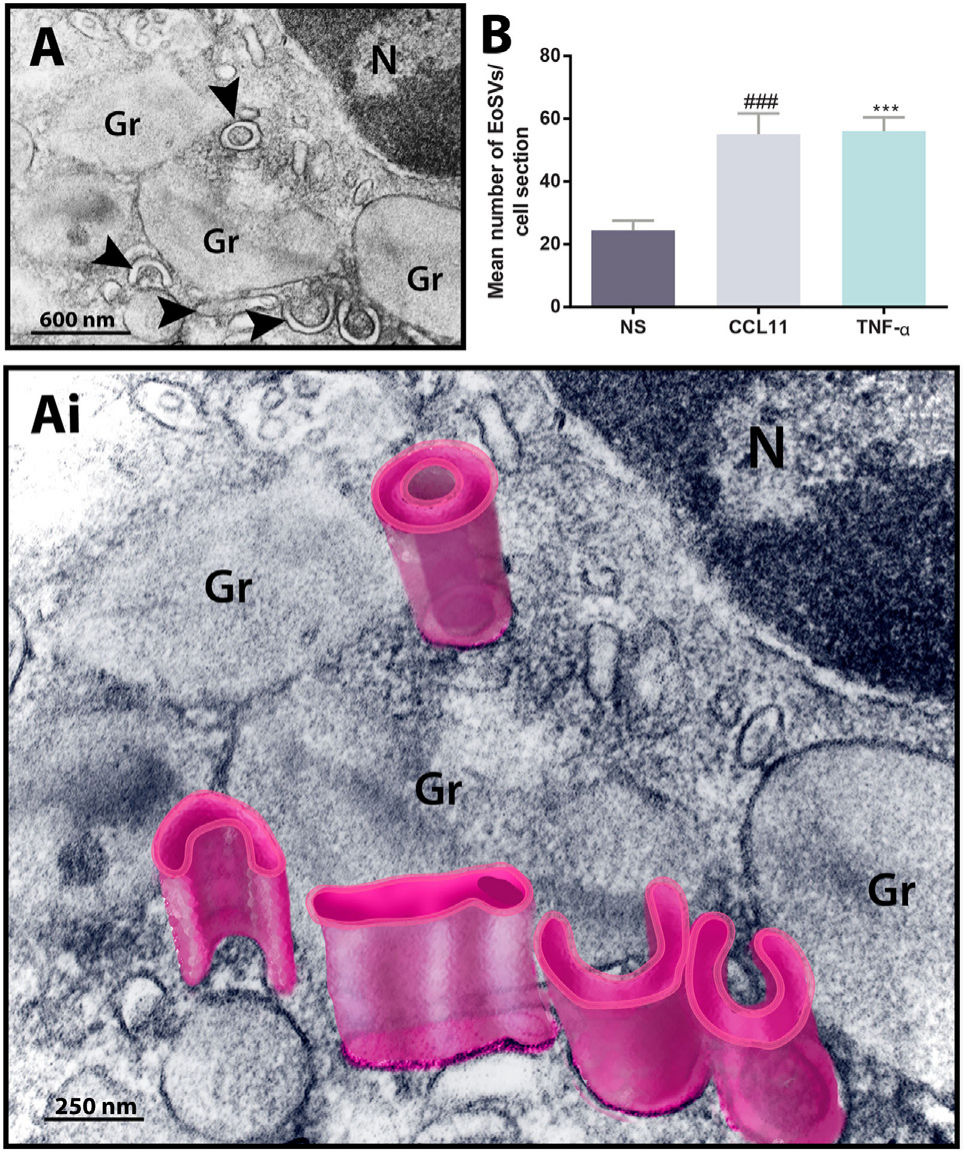
Ultrastructure of eosinophil sombrero vesicles (EoSVs). **(A,Ai)** EoSVs are large tubular vesicles which are seen in cross-sectional appearance in electron micrographs as curved, elongated or folded circumferential structures [arrowheads in **(A)** and highlighted in pink in **(Ai)**]. EoSVs are derived from and interact with secretory granules (Gr). These vesicles are frequently observed around or in contact with granules in process of emptying **(A,Ai)**, and their number increases in response to cell activation with stimuli such as CCL11 and tumor necrosis factor alpha (TNF-α) **(B)**. Data represent mean ± SEM. Electron micrographs (*n* = 30) from unstimulated and stimulated cells were evaluated, and the numbers of EoSVs (*n* = 1,357) were counted in each cell section. NS, not stimulated. ^###^*P* = 0.0004 versus NS group; ***P = 0.0003 versus NS group.

To get more evidence of eosinophil activation, we also analyzed the population of cytoplasmic EoSVs in samples conventionally prepared for EM. Because these tubular carriers have a large size and typical morphology, seen as elongated, curved, or folded circumferential structures (12, 22) (Figures 2A,Ai), the number of these carriers can be easily enumerated by means of single-cell analyses. Quantitative TEM revealed that both CCL11 and TNF-α-activated eosinophils significantly amplified the numbers of cytoplasmic EoSVs compared to the unstimulated group (Figure 2B), confirming that this vesicular system is actively formed in response to cell activation with inflammatory mediators (12, 16).

### Secretory Granules Are Consistently Labeled for IFN-γ in Both Unstimulated and Stimulated Cells

**Figure 3:**
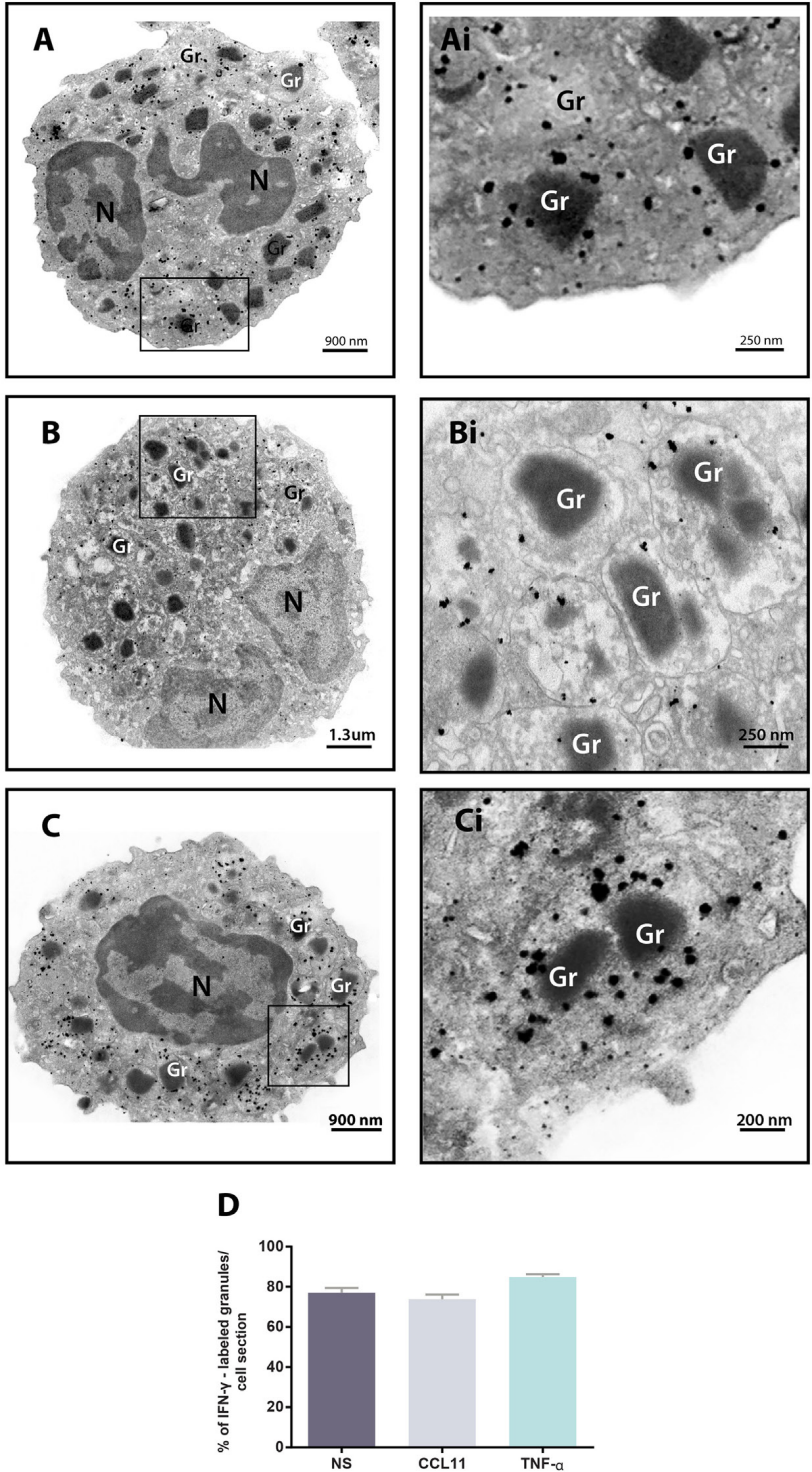
Immunolocalization of IFN-γ in unstimulated and stimulated human eosinophils. **(A–C)** Single-cell analyses at high-resolution reveal a robust labeling of IFN-γ within secretory granules of unstimulated **(A)** and CCL11- **(B)** and tumor necrosis factor alpha (TNF-α)- **(C)** activated eosinophils. The boxed areas in **(A–C)** are shown at higher magnification in **(Ai–Ci)**. Gr, secretory granule. N, nucleus. **(D)** More than 70% of the granules were positive for IFN-γ. Data represent mean ± SEM. NS, not stimulated. Cells were isolated from the peripheral blood, stimulated or not with CCL11 or TNF-α, and prepared for pre-embedding immunanogold EM.

We next performed pre-embedding immunonanogold EM for single-cell investigation of subcellular compartments labeled for IFN-γ using a protocol developed by us for optimal antigen and cell morphology preservation (17). A total of 93 cells were randomly analyzed. We found positive sites for IFN-γ in 100% of the cells, regardless of whether the eosinophils were stimulated or not, while control cells, for which the primary antibody was replaced by an irrelevant antibody, were negative or showed negligible labeling (Figure S1 in Supplementary Material). Secretory granules were remarkably labeled for IFN-γ. By using software for enumerating these granules, we detected that most of them (more than 70%) in each cell section were positive for IFN-γ in both unstimulated and stimulated cells (Figures 3A–C). Unstimulated cells showed 33.2 ± 1.7 IFN-γ-positive granules/cell section corresponding to 77.1 ± 2.4% of the total number of granules (mean ± SEM, *n* = 29 cells) whereas CCL11- and TNF-α-activated cells had 32.4 ± 1.6 and 37.6 ± 2.0 IFN-γ-positive granules/cell section, respectively, corresponding to 73.7 ± 2.3 and 84.9 ± 1.3% of the total number of granules (mean ± SEM, *n* = 34 and 30 for CCL11 and TNF-α) (Figure 3D). Labeling was seen within the granules (matrix) and also at the granule limiting membranes (Figures 3Ai–Ci).

### Identification of a Vesicular Trafficking of IFN-γ Within Human Eosinophils

**Figure 4:**
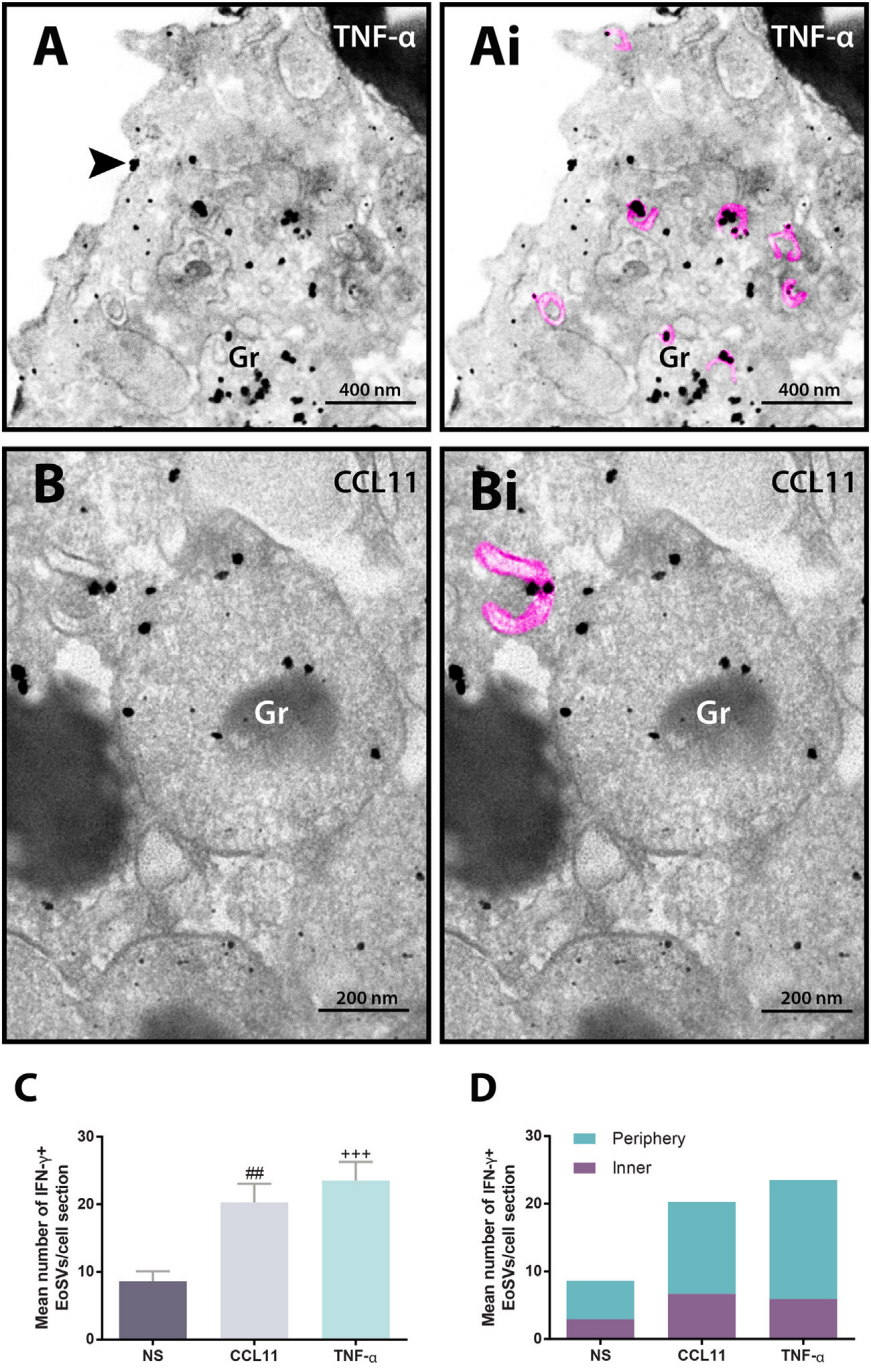
Vesicular trafficking of IFN-γ in the cytoplasm of human eosinophils. IFN-γ-positive Eosinophil Sombrero Vesicles (EoSVs) [highlighted in pink in **(Ai)** and **(Bi)**] are seen in activated cells after stimulation with tumor necrosis factor alpha (TNF-α) **(A)** or CCL11 **(B)**. Note that labeling is mostly associated with the vesicle membranes. EoSVs carrying IFN-γ are distributed in the peripheral cytoplasm **(A)** and in close association with secretory granules (Gr) undergoing release of their contents **(A,B)**. **(C)** After stimulation, the total numbers of IFN-γ-positive EoSVs significantly increased. **(D)** Most EoSVs immunolabeled for IFN-γ were observed in the cell periphery. Arrowhead in **(A)** indicates extracellular release of IFN-γ. Data represent mean ± SEM. NS, not stimulated. The numbers of labeled and not labeled EoSVs (*n* = 1,357 vesicles) were counted in cell sections (*n* = 30). ^##^*P* = 0.0051 versus NS group; ^+++^*P* = 0.0004 versus NS group. Cells were isolated from the peripheral blood, stimulated or not with CCL11 or TNF-α, and prepared for pre-embedding immunanogold EM.

In addition to immunolocalization in secretory granules, our immunonanogold EM approach revealed clear labeling for IFN-γ on EoSVs (Figure 4). Vesicles carrying IFN-γ were seen around or/and in contact with secretory granules and also distributed in the cytoplasm (Figures 4A,B). Immunolabeling was markedly associated with the vesicle membranes (Figures 4Ai,Bi). We next evaluated whether the population of IFN-γ-positive vesicles changed in the stimulated groups compared to unstimulated eosinophils. Our quantitative analyses revealed that the numbers of IFN-γ-positive EoSVs increased in response to cell activation. While the unstimulated group had 8.6 ± 1.5 of IFN-γ-positive EoSVs per cell section (mean ± SEM, *n* = 10 cells), these numbers were 20.3 ± 2.7 (mean ± SEM, *n* = 10 cells) and 23.5 ± 2.8 (mean ± SEM, *n* = 10 cells) for CCL11- and TNF-α-stimulated groups, respectively (Figure 4C). This means that the numbers of EoSVs transporting IFN-γ per cell section had more than 200% increase in the cytoplasm in response to cell activation.

**Figure 5:**
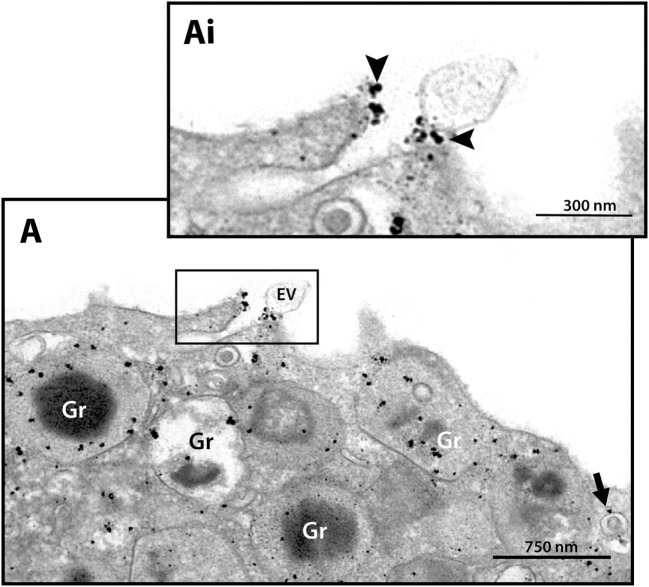
Extracellular immunodetection of IFN-γ in human eosinophils. A representative electron micrograph shows IFN-γ at the cell surface (arrowheads). A labeled extracellular vesicle (EV) is also observed. The arrow indicates an IFN-γ-positive EoSV. Note the clear labeling at its delimiting membrane. Secretory emptying granules (Gr) are prominently labeled. The boxed area in **(A)** is shown in **(Ai)** in higher magnification. Eosinophils were isolated from the peripheral blood, stimulated with CCL11 and prepared for pre-embedding immunanogold EM.

Next, we hypothesized that if a specific vesicular system is actively trafficking IFN-γ from the secretory granules for extracellular release in response to cell activation, these vesicles would present a differential distribution in the cytoplasm. We then quantitated the numbers of IFN-γ-labeled EoSVs per cytoplasm region and in fact found that these numbers were increased in the peripheral cytoplasm (within 1.0 µm of the plasma membrane) compared to the adjacent cytoplasmic area deeper in the cell (Figure 4D). Moreover, we found extracellular labeling for IFN-γ at the cell surface, indicative of cytokine release (Figures 4A and 5). Extracellular vesicles (EVs) positive for IFN-γ were occasionally found (Figure 5).

## Discussion

Precise immunolocalization of cytokines in cells from the immune system, such as eosinophils, is of critical importance to understand the capabilities of these cells during immune responses. These data presented in this work demonstrate, for the first time, that IFN-γ is mobilized and traffics in granule-derived vesicles upon cell activation. Our data also provide direct evidence that IFN-γ is constitutively stored in human eosinophils.

Here, we show that unstimulated eosinophils have a substantial pool of IFN-γ compartmentalized within secretory granules, in accordance with previous work (9). In fact, cytokines and other immune mediators are mostly stored within human eosinophils as intragranular preformed pools, from where they are mobilized, transported across the cytoplasm, and released [reviewed in Ref. (10, 26)]. Thus, eosinophil cytokines represent a group of unconventionally secreted proteins, which are released from secretory granules *via* mechanisms operating independently of the endoplasmic reticulum/Golgi complex. Complete characterization of this nonclassical protein export route and its molecular machinery is still lacking mainly due to technical challenges such as inadequate preservation of intracellular membranous microdomains and an inability of antibodies to access them (17). With the use of technical improvements and single-cell analyses at high resolution by immunonanogold EM, the intricate secretory pathway within human eosinophils has been uncovered [reviewed in Ref. (10, 26)]. Our protocol for ultrastructural detection of IFN-γ employs very small gold particles (1.4 nm in diameter) covalently conjugated with Fab fragments, which are only one-third the size of a whole IgG molecule (17). These probes improve antibody penetration and provide effective labeling of small compartments (27, 28)^.^ Moreover, we performed immunolabeling before any EM procedure, which is adequate for optimal preservation of certain types of antigens such as cytokines (17).

The present work expands our understanding that a large population of membrane-bound tubular vesicles (EoSVs) is involved in the intracellular transport of granule-stored cytokines in human eosinophils. Previous works from our group have demonstrated that EoSVs are also shuttling IL-4 (12) and other immune mediators such as major basic protein, a cationic protein that is the main constituent of the crystalloid cores of specific granules (14) and CD63, a member of the transmembrane-4 glycoprotein superfamily (tetraspanins), which is considered a marker for cell secretion (16). EoSVs are, therefore, directly involved in the traffic of granule-derived products within human eosinophils.

To elicit eosinophil secretion, we stimulated the cells with CCL11 or TNF-α. Both cytokines are well-known inducers of eosinophil activation and release of specific products from eosinophil secretory granules (9, 15, 18, 19, 21), including secretion of IFN-γ (9). In fact, by investigating the differential patterns of cytokine release from human eosinophils, we found that a large quantity of IFN-γ was secreted in response to Th1, Th2, and inflammatory stimuli (9). TNF-α proved to be a vigorous stimulus, triggering secretion of IL-4, IL-6, and IFN-γ from these cells (9). Moreover, TNF-α was considered central for IFN-γ-induced secretion of Th1-type chemokines and to increase IL-4-induced secretion of Th2-type chemokines by human eosinophils (21).

One interesting aspect of eosinophil activation is the increase in numbers of EoSVs in the cytoplasm [reviewed in Ref. (29)]. Both CCL11 and TNF-α led to the formation of EoSVs. It is now clear that EoSVs are useful morphological markers for human eosinophil activation [reviewed in Ref. (29)] being found in increased numbers even within naturally activated eosinophils from patients with hypereosinophilic syndrome when compared to normal donors (14).

The present work not only confirms the increase of the total number of cytoplasmic EoSVs but also demonstrates that the number of IFN-γ-positive EoSVs is augmented in response to cell activation (Figure 4C). The identification of this event was made possible with single-cell analyses at high resolution, thus highlighting this tool to advance our understanding of immune cell biology. A single-cell investigation also enabled definition of the distribution of EoSVs involved in the transport of IFN-γ across the cytoplasm. We found more labeled vesicles in the peripheral cytoplasm (within a band of just 1 µm wide from the plasma membrane) (Figure 4D) compared to the rest of the cytoplasm. This differential distribution denotes the occurrence of a robust traffic of this cytokine from secretory granules to the cell periphery for extracellular release. In fact, our approach captured IFN-γ at the cell surface (Figures 4A and 5). Interestingly, EVs, very small membrane-delimited vesicles, labeled for IFN-γ were also seen (Figure 5). In a previous work, we demonstrated that human eosinophils activated with inflammatory stimuli such as CCL11 and TNF-α release EVs, although their cargos were not addressed (30). In the present study, we provide evidence that EVs may be trafficking cytokines as previously suggested and potentially contributing to inflammation [reviewed in Ref. (31)]. In fact, single-cell analyses using immunonanogold EM may be useful to further investigate IFN-γ trafficking and release during different inflammatory conditions, including within the context of tissue inflammation, not only in human eosinophils but also in other cells from the immune system. This is important to understand the complex role of IFN-γ during inflammation.

Finally, single-cell imaging of IFN-γ-positive EoSVs drew our attention to the fact that immunolabeling was preferentially detected at vesicle membranes (Figures 4A,B and 5). EoSVs act as suitable intracellular carriers to accommodate membrane-bound proteins because of their curved and elongated morphology with a higher surface-to-volume ratio (22). The membrane-associated transport of IFN-γ likely reflects the presence of IFNγR alpha chains on EoSV membranes. It is known that intracellular receptors specific for different eosinophil-derived mediators are expressed in human eosinophils (13). The recognition of pools of ligand-binding cytokine receptor chains such as IL-4R alpha (13) on eosinophil secretory granules uncovers mechanisms for selective chaperoned release of cytokines. Secretory granules isolated from human eosinophils likewise express domains of IFN-γ receptors alpha chain on their membranes (32), and it is probable that EoSVs arising from granules are also carrying these receptors. However, the presence of IFN-γ receptors on membranes of EoSVs remains to be addressed in future studies.

Taken together, our findings at a single-cell level identify subcellular compartments within human eosinophils involved in the storage and trafficking of IFN-γ, with detection of a robust granule-derived vesicular transport for this cytokine in response to cell activation. This is important to understand how IFN-γ is trafficked and secreted during inflammatory responses.

## Ethics Statement

This study was carried out in accordance with the ethical principles taken from the Declaration of Helsinki and written informed consent was obtained from donors. Institutional Review Board (IRB) approval was obtained from the Beth Israel Deaconess Medical Center Committee on Clinical Investigation (Boston, MA, USA).

## Author Contributions

RM provided the study conception and design, and performed experiments. RM, LS, and PW provided critical editing of the manuscript. LC performed TEM analyses, acquired and analyzed the data. KB prepared and edited the figures. All authors contributed in part to writing and editing the manuscript and approved the final version.

## Conflict of Interest Statement

The authors declare that the research was conducted in the absence of any commercial or financial relationships that could be construed as a potential conflict of interest.
