# Supplementary material for: Single-Cell Analyses of Human Eosinophils at High Resolution to Understand Compartmentalization and Vesicular Trafficking of Interferon-Gamma
Source: Front Immunol. 2018 Jul 9;9:1542. doi: 10.3389/fimmu.2018.01542 (PMC6046373; doi:10.3389/fimmu.2018.01542)
Supplement: Supplementary file 1 [file Image_1.PDF]

Supplementary Material

Single-cell analyses of human eosinophils at high resolution to understand compartmentalization and vesicular trafficking of interferon-gamma

Lívia A. S. Carmo[1], Kennedy Bonjour[1], Lisa A. Spencer[2], Peter F. Weller[2] and Rossana C. N. Melo[1,2*]

[1] Laboratory of Cellular Biology, Department of Biology, Federal University of Juiz de Fora, UFJF, 36036-900, Juiz de Fora, MG, Brazil.

[2]Department of Medicine, Beth Israel Deaconess Medical Center, Harvard Medical School, Boston, Massachusetts 02215, USA

*Correspondence:
Dr. Rossana C. N. Melo e-mail: rossana.melo@ufjf.edu.br

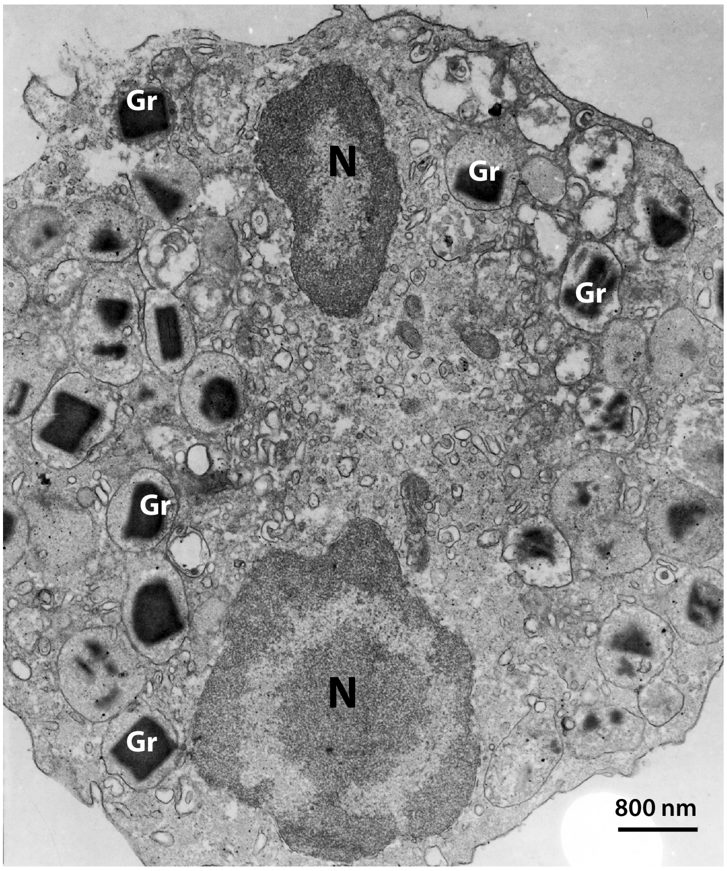

Supplementary Figure 1 Representative electron micrograph from a CCL11-stimulated human eosinophil in which the primary antibody was replaced by an irrelevant antibody. Eosinophils were isolated from the peripheral blood, stimulated or not with CCL11 or TNF-α and prepared for pre-embedding immunanogold EM. Gr, secretory granule; N, nucleus.
